# Colon and lung cancer classification from multi-modal images using resilient and efficient neural network architectures

**DOI:** 10.1016/j.heliyon.2024.e30625

**Published:** 2024-05-03

**Authors:** A. Hasib Uddin, Yen-Lin Chen, Miss Rokeya Akter, Chin Soon Ku, Jing Yang, Lip Yee Por

**Affiliations:** a Department of Computer Science and Engineering, Khwaja Yunus Ali University, Enayetpur, Chouhali, Sirajganj, 6751, Bangladesh; b Department of Computer Science and Information Engineering, National Taipei University of Technology, Taipei, 106344, Taiwan; c Department of Computer Science, Universiti Tunku Abdul Rahman, Kampar, 31900, Malaysia; d Department of Computer System and Technology, Faculty of Computer Science and Information Technology, Universiti Malaya, 50603, Kuala Lumpur, Malaysia

**Keywords:** Dense neural networks (DNN), Cancer image classification, Multi-modal network, Histopathological imaging, CT-Scan imaging, Lung cancer, Colon cancer

## Abstract

Automatic classification of colon and lung cancer images is crucial for early detection and accurate diagnostics. However, there is room for improvement to enhance accuracy, ensuring better diagnostic precision. This study introduces two novel dense architectures (D1 and D2) and emphasizes their effectiveness in classifying colon and lung cancer from diverse images. It also highlights their resilience, efficiency, and superior performance across multiple datasets. These architectures were tested on various types of datasets, including NCT-CRC-HE-100K (set of 100,000 non-overlapping image patches from hematoxylin and eosin (H&E) stained histological images of human colorectal cancer (CRC) and normal tissue), CRC-VAL-HE-7K (set of 7180 image patches from N = 50 patients with colorectal adenocarcinoma, no overlap with patients in NCT-CRC-HE-100K), LC25000 (Lung and Colon Cancer Histopathological Image), and IQ-OTHNCCD (Iraq-Oncology Teaching Hospital/National Center for Cancer Diseases), showcasing their effectiveness in classifying colon and lung cancers from histopathological and Computed Tomography (CT) scan images. This underscores the multi-modal image classification capability of the proposed models. Moreover, the study addresses imbalanced datasets, particularly in CRC-VAL-HE-7K and IQ-OTHNCCD, with a specific focus on model resilience and robustness. To assess overall performance, the study conducted experiments in different scenarios. The D1 model achieved an impressive 99.80 % accuracy on the NCT-CRC-HE-100K dataset, with a Jaccard Index (J) of 0.8371, a Matthew's Correlation Coefficient (MCC) of 0.9073, a Cohen's Kappa (Kp) of 0.9057, and a Critical Success Index (CSI) of 0.8213. When subjected to 10-fold cross-validation on LC25000, the D1 model averaged (avg) 99.96 % accuracy (avg J, MCC, Kp, and CSI of 0.9993, 0.9987, 0.9853, and 0.9990), surpassing recent reported performances. Furthermore, the ensemble of D1 and D2 reached 93 % accuracy (J, MCC, Kp, and CSI of 0.7556, 0.8839, 0.8796, and 0.7140) on the IQ-OTHNCCD dataset, exceeding recent benchmarks and aligning with other reported results. Efficiency evaluations were conducted in various scenarios. For instance, training on only 10 % of LC25000 resulted in high accuracy rates of 99.19 % (J, MCC, Kp, and CSI of 0.9840, 0.9898, 0.9898, and 0.9837) (D1) and 99.30 % (J, MCC, Kp, and CSI of 0.9863, 0.9913, 0.9913, and 0.9861) (D2). In NCT-CRC-HE-100K, D2 achieved an impressive 99.53 % accuracy (J, MCC, Kp, and CSI of 0.9906, 0.9946, 0.9946, and 0.9906) with training on only 30 % of the dataset and testing on the remaining 70 %. When tested on CRC-VAL-HE-7K, D1 and D2 achieved 95 % accuracy (J, MCC, Kp, and CSI of 0.8845, 0.9455, 0.9452, and 0.8745) and 96 % accuracy (J, MCC, Kp, and CSI of 0.8926, 0.9504, 0.9503, and 0.8798), respectively, outperforming previously reported results and aligning closely with others. Lastly, training D2 on just 10 % of NCT-CRC-HE-100K and testing on CRC-VAL-HE-7K resulted in significant outperformance of InceptionV3, Xception, and DenseNet201 benchmarks, achieving an accuracy rate of 82.98 % (J, MCC, Kp, and CSI of 0.7227, 0.8095, 0.8081, and 0.6671). Finally, using explainable AI algorithms such as Grad-CAM, Grad-CAM++, Score-CAM, and Faster Score-CAM, along with their emphasized versions, we visualized the features from the last layer of DenseNet201 for histopathological as well as CT-scan image samples. The proposed dense models, with their multi-modality, robustness, and efficiency in cancer image classification, hold the promise of significant advancements in medical diagnostics. They have the potential to revolutionize early cancer detection and improve healthcare accessibility worldwide.

## 1 Introduction

Globally, cancer-related mortality is predominantly attributed to colon and lung cancer. Accurately classifying these tumors is essential for improving patient prognosis and formulating effective treatment plans. However, achieving optimal classification accuracy remains challenging, primarily due to the intricate characteristics evident in histopathology images of cancer. The motivation behind this study is rooted in the potential to significantly improve patient outcomes and streamline the diagnostic process. Accurate cancer classification can lead to timely interventions and personalized treatment strategies, ultimately saving lives.

This study on colon and lung cancer classification emphasizes the diversity within these cancer types. Using a comprehensive dataset, the study builds on recent advancements in computer-assisted diagnosis specific to colon and lung tumors. Over the past years, various deep learning and machine learning methodologies have been explored to create automatic classification models. With the aid of expansive datasets derived from histopathology scans, these models aim to achieve unparalleled diagnostic precision.

In Tsai et al. [1], the authors introduced the Multi-omics Multi-Cohort Assessment (MOMA) framework, which examines the relationships between colorectal cancer (CRC) patients, their histological patterns, molecular features, and clinical profiles. MOMA effectively predicts copy number variations, overall survival, and disease-free survival in CRC patients using transparent machine learning, making it instrumental for CRC patients in determining treatment options.

In Li et al. [2], a lightweight convolutional neural network named CRCCN-Net was devised to automatically classify colorectal tissue histopathology images. Tested on two available datasets, the network showcased impressive accuracy, sensitivity, precision, and specificity. Moreover, in terms of computational efficiency and classification performance, it surpassed existing models, suggesting its potential as a diagnostic tool for colorectal cancer.

The study by Moyes et al. [3] offers a solution to domain shifting, a challenge in histopathological automation, using the Multi-Channel Auto-Encoder (MCAE) approach. The MCAE model surpasses prevailing techniques for identifying unknown domains through normalized feature representation evaluation.

In Naga Raju and Srinivasa Rao [4], a refined hybrid of deep learning and machine learning for diagnosing colon and lung cancer was presented. This method leverages digital histopathological images and yields enhanced classification results, contributing to the evolution of precise and automated cancer screening systems.

Srivastava et al. [5] aimed to boost the classification performance for colon and lung cancer using the LC25000 histopathology image set. The proposed ensemble method is based on Condorcet's jury theorem. Experimental results, with a precision of 99.78 % and 99.88 % for the ensemble models, clearly outperformed contemporary techniques.

Diao et al. [6] introduced a deep learning system using multiple magnifications (DSML) for accurate histopathology image classification. This method not only surpasses existing techniques but also elucidates the efficiency of multi-magnification learning.

Pradhan and Sahu [7] proposed a unique model for the automatic classification of lung histopathology images, integrating color normalization, SDREL-based cancer segmentation, and feature extraction from Alexnet and GLCM. With a commendable score of 98.50 % on the LC25000 lung histopathology dataset, the method underscores the significance of optimal feature selection.

Ram et al. [8] detailed the application of a machine learning technique named the graph-based sparse principal component analysis (GS-PCA) network for the auto-detection of malignant lesions in histological lung slides. Using methods like Support Vector Machine (SVM) classification, cascaded graph-based sparse PCA, PCA binary hashing, and block-wise histograms, the recommended method's detection accuracy clearly excels when compared to established techniques.

Reis and Turk [9] leveraged transfer learning and deep learning for nucleus detection and classification in histopathology datasets. The method achieved a 95.0 % accuracy rate on the MNIST collection of colorectal histopathology data using the DenseNet169 model. This technique holds promise for assisting medical professionals in detecting and managing colon cancer.

Rajput and Subasi [10] delved into the use of deep learning methodologies for the automated detection of lung cancer via histopathology images. By integrating a pre-support vector model, the ResNet model exhibits a high accuracy of 98.57 %, ensuring swift and accurate lung cancer diagnostics.

Rajput and Subasi [11] investigated the precise identification of colon cancer in histopathology images, deploying deep learning techniques and pretrained models. Using ResNet50, the model astonishingly achieves an accuracy of 99.8 % on test data, drastically reducing the time taken to detect colon cancer.

The above-mentioned works introduce and develop numerous excellent solutions in order to provide near-perfect performance for the classification of lung and colon cancers from histopathological images. While those works produce noteworthy solutions, they often lack multi-modality, robustness, and efficiency. Those solutions do not incorporate experiments on multiple types of cancer images but rather only deal with one type, such as histopathological cancer images. Also, to deal with the class imbalance problem, these methods often have to rely on extensive processing, requiring more resources and time. Moreover, large cancerous image datasets often demand exhaustive resources to develop and train neural architectures.

In this study, we proposed two neural network architectures that can train well on multiple types of cancer images, require minimal preprocessing to deal with imbalanced datasets, require a small amount of data to generalize on the entire dataset, and provide optimal performance.

Our contributions in this paper can be summarized as follows.

1. Multi-modality: We have proposed two models capable of classifying lung cancer images from both histopathological and CT-scan types, as well as effectively classifying both lung cancer and colon cancer from different datasets.
2. Robustness: Our models can properly classify lung and colon cancer images from both balanced and imbalanced datasets.
3. Efficiency: Our models can learn efficiently with a low number of images.

The subsequent sections of the paper cover various topics. Section 2 provides a thorough summary of the literature. Section 3 covers a brief introduction of the datasets, the methods used, the suggested methods’ architectures, and the training environments. In Section 4, the results are analyzed, current related publications are compared, and pertinent remarks are included along with illustrations of the properties of the suggested model throughout the layers. The essay concludes in Section 5, outlining potential future directions.

## 2 Related works

Sethy et al. combined AlexNet, wavelet, and a support vector machine to create a hybrid network in their article [12]. They employed the Lung and Colon LC25000 Histopathology Image Collection for this research to evaluate and train SVM classifiers. Tenfold cross-validation was used to determine the study's accuracy, which reached 99.3 %.

Hadiyoso et al. [13] proposed a deep learning technique for automatically classifying colon and lung cancer. They classified 25,000 histopathology images using the VGG16 architecture and contrast-limited adaptive histogram equalization (CLAHE). The simulation results demonstrate that the suggested method can categorize data with a potential accuracy of 98.96 %.

In their work, Wahid et al. [14] utilized three pre-trained Convolutional Neural Network (CNN) models: ResNet18, GoogLeNet, and ShuffleNet V2. They also employed the LC25000 dataset and a straightforward modified CNN model. ResNet18 achieved a precision rate of 98.82 %. ShuffleNet V2 emerged as the top model for colon data classification, with an accuracy rate of 99.87 %. Their proposed modified CNN model achieved a lung cancer accuracy of 93.02 % and a colon cancer accuracy of 88.26 %.

Iqbal et al. [15] introduced a new approach called ColonNet. They compared their proposed ColonNet model to state-of-the-art CNNs, showing that it outperformed others on the test set with an impressive F1-score of 0.96, sensitivity and specificity of 0.95, and an area under the accuracy curve of 0.95.

Al Ghamdi et al. [16] aimed to construct a transfer learning method for histological image analysis for lung and colon cancer identification. They suggested a model that combines deep convolutional recurrent neural networks with an improved ShuffleNet. Additionally, they used the Optimization of Coati and Al-Biruni Earth Radii algorithms for hyperparameter tuning. The proposed model achieved an experimental accuracy of 98.99 % on the LC25000 database.

Stephen and Sain [17] provided a neural architecture search algorithm that efficiently detects colon and lung cancers in histological images. Their method produced an accurate neural network architecture for categorization and detection of colon and lung cancers, achieving an accuracy rate of 93.91 % on the LC25000 dataset.

Kumar et al. [18] discussed colon and lung cancer detection using a traditional transfer learning approach with pre-trained CNN networks as feature extractors. The RS classifier using features from the DenseNet-121 pre-trained network outperformed all other classifiers with a precision of 98.60 %.

Hage Chehade et al. [19] employed machine learning, feature engineering, and image processing methods to classify histological images of colon and lung malignancies using data from the LC25000 dataset. They tested six models: light gradient-boosting machine (LightGBM), eXtreme gradient boosting (XGBoost), SVM, random forest (RF), linear discriminant analysis (LDA), and multi-layer perceptron (MLP). The XGBoost model achieved an F1-score and accuracy of 98.8 % and 98.6 %, respectively.

Mridha et al. [20] recommended using a deep learning framework to distinguish between five different types of lung and colon cancer cells. Their method established techniques for evaluating and analyzing histopathology images of cancer cells, with the highest testing accuracy reaching 98.3 %.

Mehmood et al. [21] proposed a highly precise computing model for the diagnosis of lung and colon cancers. They fine-tuned a pretrained neural network (AlexNet) with histology images, improving the overall accuracy from 89 % to 98.4 %.

Provat et al. [22] introduced a deep learning approach for the detection of lung and colon cancer using the LC25000 dataset. They improved accuracy and computing performance by using cyclic learning rates, and they achieved an accuracy rate of 97 % with their model.

Mohalder et al. [23] proposed a deep learning algorithm for identifying lung tumors from histopathology images based on clinical CT images from various sources. Their deep learning model achieved a precision of 95 %.

Talukder et al. [24] developed a technique for extracting a hybrid ensemble of features to distinguish between lung and colon cancer with a 99.30 % accuracy rate using histopathological LC25000 datasets.

Wadeker et al. [25] explored the combination of machine learning and medical imaging for cancer diagnosis. Their accuracy reached 97.73 % using an improved augmentation technique and a tuned VGG19 model.

Masud et al. [26] presented modern deep learning techniques for distinguishing between benign and malignant lung and colon tumor types using histology images. Their maximum accuracy for recognizing malignant tissues was 96.33 %.

Adu et al. [27] introduced a novel dual horizontal squash capsule network (DHS-CapsNet) for histological image-based classification of lung and colon cancers, achieving an accuracy of 99.23 %.

Ibrahim et al. [28] established an AI classification strategy to distinguish between three malignant and two benign forms of lung and colon tissues. Their accuracy rating reached 99.5 % using deep learning (DL) techniques and image enhancement.

Karim et al. [29] proposed a deep learning strategy based on CNN for the classification of lung tissue samples, achieving a training accuracy of 98.15 % and a validation accuracy of 98.07 %.

Hossain et al. [30] recommended using convolutional networks with digital pathology to aid in colon cancer diagnosis, achieving an accuracy rate of 94 % for distinguishing between tissue cells, benign colon tissues, and colon cancer tissues in histopathological images.

Table 1 summarizes the research efforts in the field of colon and lung cancer identification through machine learning and deep learning techniques. Sethy et al. [12] combined AlexNet and SVM, achieving an impressive accuracy of 99.3 %. Methods encompassed a wide range, from hybrid networks to image enhancements using VGG16 and CLAHE, as seen in the work of Hadiyoso et al. [13]. Other researchers, such as Iqbal et al. [15], focused on achieving higher F1-scores, while others emphasized transfer learning, as exemplified by the studies conducted by Kumar et al. [18] and Al Ghamdi et al. [16]. Neural architecture searches were employed by Stephen and Sain [17].

**Table 1:** Synthesis and summary table for the related work on the Lung and Colon Cancer Histopathological Image (LC25000) dataset.

Ref	Method/Model	Dataset	Main Achievement	Accuracy (%)	Limitation
Sethy et al. [12]	Hybrid (AlexNet, SVM)	LC25000	Hybrid network	99.30	i. Only works on histopathological images. ii. Tested only on one dataset.
Hadiyoso et al. [13]	VGG16 with CLAHE	LC25000	Image enhancement	98.96	
Wahid et al. [14]	Various CNNs	LC25000	Multiple models	Up to 99.87	
Iqbal et al. [15]	ColonNet	LC25000	High F1-score	96.31	
Al Ghamdi et al. [16]	ShuffleNet, DCRNN	LC25000	Transfer learning	98.99	
Stephen and Sain [17]	Neural Arch Search	LC25000	Neural net search	93.91	
Kumar et al. [18]	Transfer Learning	LC25000	Feature extraction	98.60	
Hage Chehade et al. [19]	Various ML models	LC25000	Multiple models	Up to 99	
Mridha et al. [20]	DL Framework	LC25000	5 types of cells	98.30	
Mehmood et al. [21]	Modified AlexNet	LC25000	Fine-tuned model	98.40	
Provat et al. [22]	Custom CNN	LC25000	Cyclic learning rates	97.00	
Mohalder et al. [23]	DL Model	LC25000	CT images	95.00	
Talukder et al. [24]	Ensemble	LC25000	Hybrid features	99.30	
Wadeker et al. [25]	VGG19	LC25000	Image augmentation	97.73	
Masud et al. [26]	DL Framework	LC25000	5 types of tumors	96.33	
Adu et al. [27]	DHS-CapsNet	LC25000	Capsule Network	99.23	
Ibrahim et al. [28]	Custom NN with CLAHE	LC25000	Image enhancement	99.50	
Karim et al. [29]	Custom CNN	LC25000	3 types of lung tissue	Up to 98.15	
Hossain et al. [30]	CAD	LC25000	Digital pathology	94.00	

Several other techniques included fine-tuning existing networks like AlexNet, as demonstrated by Mehmood et al. [21], utilizing cyclic learning rates for custom CNNs, as explored by Provat et al. [22], and introducing novel networks like DHS-CapsNet, as proposed by Adu et al. [27]. The choice of datasets varied across studies, with the LC25000 dataset being a common selection among many researchers. Notably, these methodologies consistently achieved impressive accuracy rates, often surpassing the 98 % threshold.

## 3 Methods

**Fig. 1:**
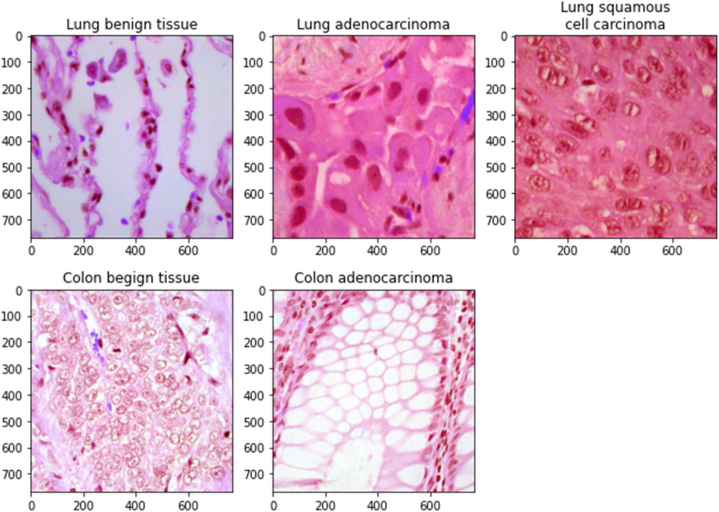
Example images from the LC25000 histopathological dataset.

The statistical summary of the NCT-CRC-HE-100K, CRC-VAL-HE-7K, LC25000, and IQ-OTHNCCD datasets is provided in Table 2. Fig. 1 displays examples of photos from each class in the LC25000 dataset. This dataset is the augmented and processed version of the original dataset [31]. It has a balanced number of images in each of its five categories. Because of the augmentation and data balance, it is quite easy to achieve high performance on this dataset. Table 3 presents various scenarios that were employed in this study to demonstrate the multi-modality, robustness, and efficiency of the proposed D1 and D2 models across diverse datasets.

**Table 2:** Statistical summary of the Lung and Colon Cancer Histopathological Image (LC25000), set of 100,000 non-overlapping image patches from hematoxylin & eosin (H&E) stained histological images of human colorectal cancer (CRC) and normal tissue (NCT-CRC-HE-100K), set of 7180 image patches from N = 50 patients with colorectal adenocarcinoma, no overlap with patients in NCT-CRC-HE-100K (CRC-VAL-HE-7K), and Iraq-Oncology Teaching Hospital/National Center for Cancer Diseases (IQ-OTHNCCD) datasets.

Dataset	Cancer-type	Image-type	Dimension	Classes	Total images	Balanced	Additional Information
LC25000 [31]	Lung and Colon	Histo-pathological	768 x 768	3 classes (lung): Benign (Lung_N), Adenocarcinoma (Lung_ACA), Squamous cell carcinomas (Lung_SCC) 2 classes (colon): Benign (Colon_N), Adenocarcinoma (Colon_ACA)	Total 25000: Lung_N (500), Lung_ACA (500), Lung_SCC (500), Colon_N (500), Colon_ACA (500)	Yes	Real size: 1024 x 768 pixels
NCT-CRC-HE-100K [32]	Colon	Histo-pathological	224 x 224	9 classes: Adipose (ADI), Background (BACK), Debris (DEB), Lymphocytes (LYM), Mucus (MUC), Smooth muscle (MUS), Normal colon mucosa (NORM), Cancer-associated stroma (STR), Colorectal adenocarcinoma epithelium (TUM)	Total 100,000: ADI (10,407), BACK (10,566), DEB (11,512), LYM (11,557), MUC (8,896), MUS (13,536), NORM (8,763), STR (10,446), TUM (14,317)	No	Microns Per Pixel (MPP): 0.5
CRC-VAL-HE-7K [32]	Colon	Histo-pathological	224 x 224		Total 7180: ADI (1,338), BACK (847), DEB (339), LYM (634), MUC (1,035), MUS (592), NORM (741), STR (421), TUM (1,233)	No	Microns Per Pixel (MPP): 0.5
IQ-OTHNCCD [33]	Lung	CT-scan	512 x 512	3 classes: Benign (BN), Malignant (MG), Normal (N)	BN (120), MG (561), N (416)	No	The CT protocol includes the following parameters: 120 kV, a slice thickness of 1 mm, a window width ranging from 350 to 1200 HU, and a window center from 50 to 600 for reading purposes.
CRC-7K-Down [customized by the authors]	Colon	Histo-pathological	224 x 224	Same as CRC-VAL-HE-7K	Total 3051: ADI (339), BACK (339), DEB (339), LYM (339), MUC (339), MUS (339), NORM (339), STR (339), TUM (339)	Yes	
CRC-7K-Down-MUS.STRx3 [customized by the authors]	Colon	Histo-pathological	224 x 224		Total 4407: ADI (339), BACK (339), DEB (339), LYM (339), MUC (339), MUS (1,017), NORM (339), STR (1,017), TUM (339)	No

**Table 3:** Different scenarios were applied in this study to prove the multi-modality, robustness, and efficiency of the proposed D1 and D2 models on varied datasets.

Scenario	Train Set	Test Set	Proposed Model	Aim to Prove
1	LC25000 (10-fold cross-validation)		D1	Superiority in terms of classifying lung and colon cancer from histopathological images
2	80 % of IQ-OTHNCCD	20 % of IQ-OTHNCCD	D1	Multi-modality and Robustness
3			D2	
4			Ensemble of D1 and D2	
5	10 % of LC25000	90 % of LC25000	D1	Efficiency and Robustness
6			D2	
7	80 % of NCT-CRC-HE-100K	20 % of NCT-CRC-HE-100K	D1	
8	30 % of NCT-CRC-HE-100K	70 % of NCT-CRC-HE-100K	D2	
9	10 % of NCT-CRC-HE-100K	CRC-VAL-HE-7K	D1	
10			D2	
11	CRC-VAL-HE-7K	NCT-CRC-HE-100K	D2	
12	CRC-7K-Down		D2	
13	CRC-7K-Down-MUS.STRx3		D1	
14			D2	

### 3.1 Proposed model

The proposed neural network architectures, models D1 and D2, follow a similar structure with slight variations in the number of neurons and layers. Both models start with pre-trained feature extraction using DenseNet201 or ResNet50 trained on the ImageNet dataset.

**Fig. 2:**
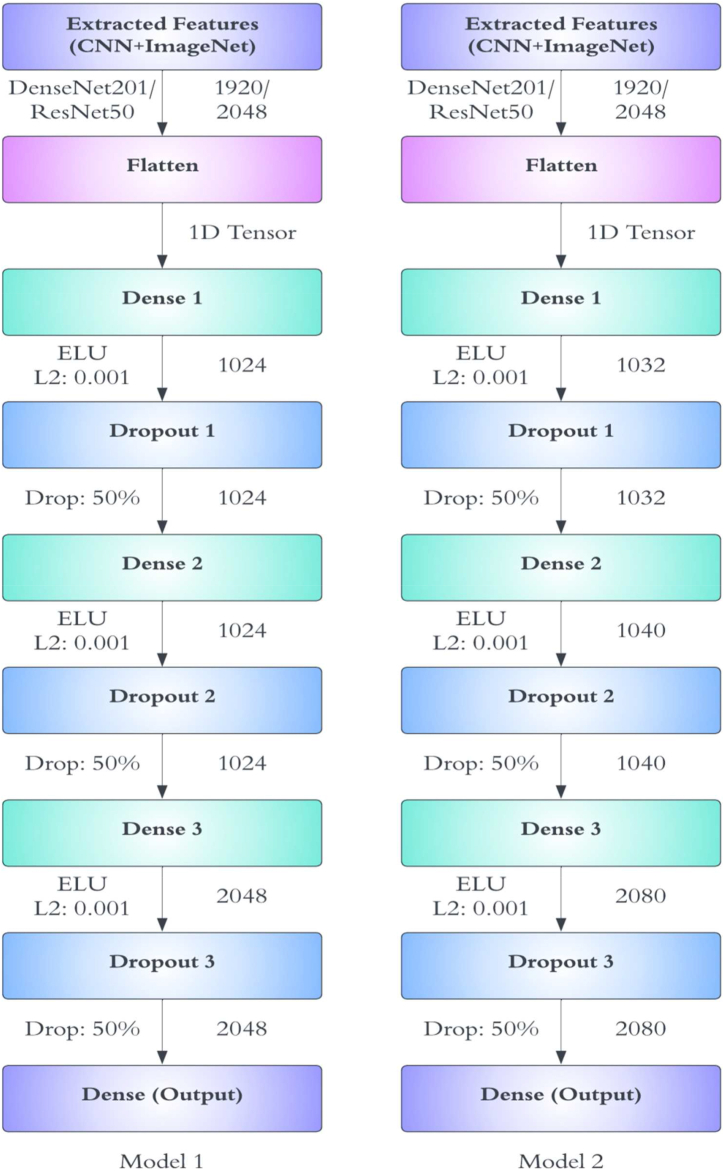
Architectures of the proposed model 1 (D1) and model 2 (D2).

Fig. 2 displays the architectures of the proposed models, which can be described as follows.

● Input Layer:
• Extracted features from DenseNet201 or ResNet50
• DenseNet201 has 1920 filters, while ResNet50 has 2048 filters.
● Flatten Layer:
• It converts the extracted features into a 1D tensor for further processing.
● Dense Layers:
• Dense 1:
  o Activation Function: ELU (Exponential Linear Unit)
  o L2 regularization: 0.001
  o Number of neurons:
  o Model 1: 1024 neurons (D1) or 1032 neurons (D2)
  o Dropout Rate: 50 %
• Dense 2:
  o Activation Function: ELU
  o L2 regularization: 0.001
  o Number of neurons:
  o Model 1: 1024 neurons (D1) or 1040 neurons (D2)
  o Dropout Rate: 50 %
• Dense 3:
  o Activation Function: ELU
  o L2 regularization: 0.001
  o Number of neurons:
  o Model 1: 2048 neurons (D1) or 2080 neurons (D2)
  o Dropout Rate: 50 %
● Output Layer:
• Dense (Output):
  o Activation Function: Softmax
  o Neurons: The number of neurons in this layer equals the number of classes in the dataset, generating class probabilities using the softmax function.

Both models (D1 and D2) consist of multiple dense layers with ELU activation functions, L2 regularization, and dropout layers to prevent overfitting. Model D2 generally has slightly more neurons in each dense layer compared to D1, potentially allowing it to capture more complex patterns at the expense of increased computational complexity and memory requirements. The final output layer utilizes softmax activation to provide class probabilities for the classes in the dataset. These architectures were constructed to be well-structured, balancing between complexity and regularization to potentially handle various classification tasks efficiently.

*DenseNet201:* The proposed models prominently feature DenseNet201 as an option for pre-trained feature extraction. This architecture, introduced by Huang et al. [34], departs from the traditional convolutional neural network (CNN) structure by employing a dense connectivity pattern between layers.

In a standard CNN, information flows forward through the network, with each layer receiving input only from the previous layer. DenseNet201 deviates from this approach by establishing dense connections between all layers within a certain number of layers apart. This strategy fosters feature reuse, where each layer receives not only the original input but also feature maps from preceding layers. This dense connectivity is believed to encourage better gradient propagation and alleviate the vanishing gradient problem, a common hurdle in deep neural networks.

DenseNet201 achieves its dense connectivity through the use of dense blocks. These blocks consist of multiple convolutional layers stacked together in a dense fashion, as described earlier. Within a dense block, feature maps from all preceding convolutional layers within the block are concatenated as input to the subsequent layer. This concatenation allows the network to exploit feature maps from various depths, potentially leading to richer feature representations.

Overall, DenseNet201's dense connectivity pattern and feature reuse strategy offer an alternative approach to traditional CNN architectures. The use of dense blocks and feature concatenation is believed to improve feature propagation and potentially lead to better performance, particularly in deeper networks.

*ResNet50:* Again, the proposed models also offer ResNet50 as a pre-trained feature extractor. This architecture, introduced by He et al. [35], tackles the degradation problem that can occur in deep neural networks.

Training deep neural networks can be challenging due to the vanishing gradient problem. In traditional CNNs, gradients used to update network weights tend to diminish as they backpropagate through many layers. This can make it difficult for the network to learn effectively in deeper architectures.

ResNet50 addresses this issue by incorporating residual learning blocks. These blocks introduce shortcut connections that allow the original input to be directly added to the output of a convolutional layer within the block. This creates a path for gradients to flow unobstructed, facilitating learning even in very deep networks.

ResNet50 utilizes multiple residual learning blocks stacked together. Each block may perform operations like convolution, batch normalization, and activation functions. The shortcut connection within the block enables the network to learn the residual mapping, the difference between the input and output of the convolutional layers. This residual learning is believed to be easier for the network to optimize compared to learning the entire transformation directly.

In essence, ResNet50's residual learning blocks with shortcut connections help alleviate the vanishing gradient problem, allowing for effective training of deep convolutional neural networks. This approach has demonstrably improved performance on various image recognition tasks.

*Convolutional Neural Network (CNN):* CNNs draw inspiration from the biological structure of the visual cortex in the mammalian brain [36]. The visual cortex processes visual information in a hierarchical manner, with neurons responding to specific features like edges, lines, and shapes in different regions. Similarly, CNNs employ convolutional layers with learnable filters that detect these low-level features in the input image.

Following the convolutional layers, pooling layers are often used to downsample the extracted features. This reduces the dimensionality of the data while retaining the most important information. Subsequent convolutional layers can then build upon these lower-level features, progressively extracting more complex and abstract representations.

The final stages of a typical CNN architecture typically involve fully-connected layers. These layers operate similarly to traditional neural networks, processing the flattened output from the convolutional layers and making final classifications based on the learned features.

CNNs offer several advantages over traditional neural networks for image processing tasks. Their ability to learn spatial features directly from the input data makes them highly effective in tasks like object detection and image classification. Additionally, the use of shared weights in convolutional layers reduces the number of parameters to be learned, improving efficiency and reducing the risk of overfitting.

In conclusion, CNNs have revolutionized the field of computer vision thanks to their ability to capture spatial relationships and learn hierarchical feature representations. Their success has paved the way for significant advancements in various image-related tasks.

*Dense Layers:* The proposed models prominently feature dense layers, also known as fully-connected (FC) layers. These layers play a crucial role in transforming the extracted features from the convolutional layers into meaningful outputs for tasks like classification.

Unlike convolutional layers that operate on spatial data, dense layers treat the input as a one-dimensional vector [37]. This is achieved through a flattening layer that transforms the multi-dimensional feature maps from the convolutional layers into a single long vector. Each element in this vector represents an activation from a specific neuron in the previous layer.

The core principle behind dense layers lies in their full connectivity. Unlike convolutional layers with localized filters, every neuron in a dense layer is connected to every neuron in the previous layer. This dense web of connections allows the network to combine features extracted from different parts of the input to create more complex and abstract representations.

Dense layers typically employ activation functions to introduce non-linearity into the network. This is crucial because stacked linear layers would only be able to learn linear relationships, limiting the network's ability to model complex patterns in the data. Common activation functions used in dense layers include ReLU (Rectified Linear Unit) and its variants, which introduce non-linear thresholds for activation.

The final dense layer in a classification task often uses a softmax activation function. Softmax transforms the output from the previous dense layer into a probability distribution across all possible classes. Each element in the output vector represents the probability of the input belonging to a specific class.

### 3.2 Hyper-parameters

Table 4 provides a concise overview of the hyperparameters. The descriptions of these hyperparameters are as follows.

• Batch size: The batch size refers to the quantity of data samples processed during each training cycle. In this instance, the model processes 16 samples at a time since the batch size is set to 16.
• Number of epochs: The number of epochs indicates how many times the entire dataset is passed through the model during training. In the first scenario, the model runs over the dataset five times.
• Learning rate: The learning rate controls the frequency of weight updates during training. A lower learning rate typically results in fewer updates. The learning rate is currently set to 0.00001.
• Learning rate decay: This is a small constant that ensures numerical stability and is often added to the denominator to prevent division by zero. In this case, the epsilon value is set to 1e-4, equivalent to 0.0001.
• Dropout: Dropout is a regularization technique used to prevent neural networks from overfitting. It randomly sets a portion of input units to zero during training, aiding in generalization. The dropout rate in this case is set to 0.5, meaning that 50 % of input units are randomly dropped during training.
• Loss function: The loss function assesses the model's performance and guides the training process. Categorical cross-entropy [37] is a standard loss function for multi-class classification problems. Categorical cross-entropy measures the discrepancy between the predicted probability distribution (generated by the softmax activation in the output layer) and the true probability distribution representing the correct class. Mathematically, categorical cross-entropy (H) can be expressed as:

H(p,q)=−Σ(pi*log(qi))

Where.

H(p, q) represents the categorical cross-entropy between the true probability distribution (p) and the predicted probability distribution (q).
Σ (summation) iterates over all possible classes (i).
pi represents the true probability for class i.
qi represents the predicted probability for class i (obtained from the softmax activation).

**Table 4:** Utilized hyper-parameters in this study.

Scenario	Batch Size	Image Dimension	Interpolation
1	16	256	Bi-linear
2	1	284	Bi-linear
3	1	284	Bi-linear
5	16	256	Lanczos
6	16	256	Lanczos
7	16	256	Bi-linear
8	16	256	Lanczos
9	16	224	Lanczos
10	16	224	Lanczos
11	16	224	Lanczos
12	16	224	Lanczos
13	16	224	Lanczos
14	16	224	Lanczos
**Common hyper-parameters**			
Color channel		RGB	
Re-scaling		[0–255]	
Learning rate		0.00001	
Learning decay		1e-4	
Loss		Categorical cross-entropy	
Optimizer		Adam	
Classifier		SoftMax	

Lower categorical cross-entropy values indicate better alignment between the model's predictions and the ground truth labels. This loss function guides the optimization process during training, aiming to minimize the difference between predicted and true probabilities, ultimately leading to improved classification performance.

• Activation function: The activation function determines the mathematical function applied to the output of neurons or layers in a neural network. Here, ‘ELU’ stands for Exponential Linear Unit [38], which is a specific type of activation function. This function offers several advantages over traditional activation functions like ReLU (Rectified Linear Unit). Unlike ReLU, which outputs zero for negative inputs, ELU introduces a non-zero slope for negative values. This can be mathematically expressed as:

ELU(x) = {

x if x≥0

α * (exp(x) - 1) if x < 0

}

Where.

x represents the input value to the activation function.
α (alpha) is a hyperparameter typically set to a small positive value (e.g., 1.0).

ReLU neurons can become inactive (stuck at zero) if they consistently receive negative inputs. ELU's non-zero slope for negative values helps mitigate this issue, allowing these neurons to potentially recover and contribute to the learning process.

Compared to ReLU's sharp threshold at zero, ELU's smooth transition for negative inputs allows for a more continuous gradient flow during backpropagation. This can potentially lead to faster and more stable learning in deep neural networks.

• Optimizer: The optimizer is important in selecting the method for updating the model's weights based on the gradients calculated during training. “Adam” is an optimization strategy that combines the benefits of two other well-known optimization methods: root mean square propagation (RMSprop) and the adaptive gradient method (AdaGrad). The proposed models utilize Adam (Adaptive Moment Estimation) [39], which is a widely used optimizer known for its efficiency and effectiveness in training deep neural networks. It builds upon the strengths of previous optimizers like RMSprop and AdaGrad, addressing some of their limitations.

A notable aspect of Adam is its use of adaptive learning rates for each parameter (weight) in the network (θ). Unlike traditional optimizers with a fixed learning rate, Adam dynamically adjusts the learning rate (η) for each parameter based on its historical gradients. The equations for the Adam optimizer, a popular optimization algorithm used in training neural networks, are as follows.

Initialize the parameters of the optimizer:
m = 0 (initialization of the first moment vector)
v = 0 (initialization of the second moment vector)
t = 0 (initialization of the time step)
beta_1_, beta_2_ (exponential decay rates for moment estimates)
epsilon (a small constant to prevent division by zero)
1. Compute the gradients g_t_ on the mini-batch t of the loss function L(w) with respect to parameters w.
  2. Increment time step: t = t + 1
  3. Update biased first moment estimate:

$$m_{t}={\text{beta}}_{1}\;.\;m_{t‐1}+\left(1\;‐\;{\text{beta}}_{1}\right)\;.\;g_{t}$$

4. Update biased second moment estimate:

$$v_{t}={\text{beta}}_{2}\;.\;v_{t‐1}+\left(1\;‐\;{\text{beta}}_{2}\right)\;.\;\left(g_{t}⊙g_{t}\right)$$

Here, ⊙ denotes element-wise multiplication.

5. Correct bias in the first moment:

$${\text{mhat}}_{t}=m_{t}\;/\;\left(1\;‐\;{\text{beta}}_{1}^{t}\right)$$

6. Correct bias in the second moment:

$${\text{vhat}}_{t}=v_{t}\;/\;\left(1\;‐\;{\text{beta}}_{2}^{t}\right)$$

7. Update parameters:

$$w_{t+1}=w_{t}\;‐\;\text{alpha}\;.\;{\text{mhat}}_{t}\;/\;\left(\sqrt{{\text{vhat}}_{t}}+\text{epsilon}\right)$$

Here, alpha is the learning rate.

• Combining the Best of Both Worlds: Momentum and RMSprop

Adam incorporates concepts from both momentum-based optimizers and RMSprop. The momentum term (mt) considers the past gradients of a parameter to accelerate convergence in the right direction. The RMSprop estimate (vt) addresses the issue of diminishing learning rates in AdaGrad by taking the average of squared historical gradients, preventing updates from becoming too small. Adam combines these ideas by utilizing both the historical gradient direction (mt) and the magnitude of past gradients (vt) to achieve efficient learning.

• Regularization: The proposed models incorporate L2 regularization [37] within the dense layers, as indicated by the coefficient λ (lambda) set to 0.001. This technique is a common strategy to prevent overfitting in deep neural networks.

Overfitting occurs when a neural network model becomes too focused on the specific training data it's exposed to. This can lead to the model performing well on the training data but failing to generalize accurately to unseen data. L2 regularization helps mitigate this issue.

L2 regularization works by adding a penalty term to the loss function during training. This penalty term is proportional to the sum of the squares of the weights (parameters) in the network. Mathematically, the L2 regularization term can be expressed as:

$$L2\;\text{penalty}\;\text{term}=\lambda \;*\;\Sigma \left(w^{2}\right)$$

Where.

λ (lambda) is the L2 regularization hyperparameter controlling the strength of the penalty.
Σ (summation) iterates over all weights (w) in the network.

By penalizing large weight values, L2 regularization discourages the model from becoming overly complex and relying too heavily on specific features in the training data. This encourages the model to learn more generalizable representations that perform well on both training and unseen data.

● Classifier: The proposed models employ the softmax function [37] within the final layer of the classifier. Softmax plays a crucial role in transforming the network's output into interpretable probabilities for each potential class.

Deep neural networks typically process data through multiple layers, culminating in a final layer with one neuron for each class in the classification task. These neurons generate numerical scores that reflect the network's “preference” for each class given the input. However, these scores are not directly interpretable as probabilities.

The softmax function steps in to address this by converting these raw scores into a probability distribution across all classes. Mathematically, the softmax function (σ) operates on a vector of input scores (z) and produces a vector of class probabilities (p).

p(i)=σ(z(i))=exp(z(i))/Σ(exp(z(j)))forallclassesjz(j)))forallclassesjexp

Where.

p(i) represents the probability of the input belonging to class i.
σ(z(i)) denotes the softmax function applied to the score for class i (z(i)).
exp(z(i)) calculates the exponential of the score for class i.
Σ (summation) iterates over all possible classes (j).
exp(z(j)) calculates the exponential of the score for each class (j).

The softmax function ensures that the resulting probabilities adhere to several key properties: all output probabilities are non-negative, ranging from 0 to 1. The sum of the probabilities across all classes equals 1, making it a valid probability distribution. The softmax output allows for a clear interpretation of the network's confidence level for each class. The higher the probability for a particular class, the more confident the network is that the input belongs to that class.

### 3.3 Training

**Fig. 3:**
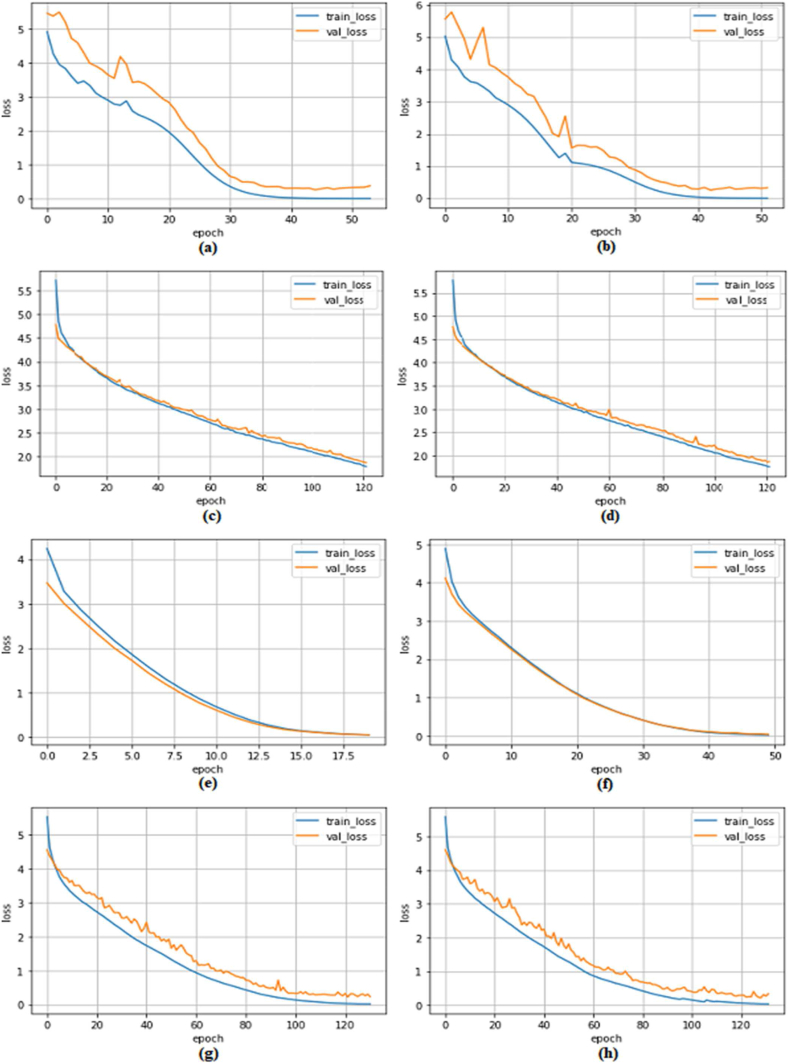
Learning curves corresponding to scenarios (a) 2, (b) 3, (c) 5, (d) 6, (e) 7, (f) 8, (g) 9, and (h) 10 from Table 3.

The models were trained on the datasets until their validation loss stopped decreasing for five consecutive epochs. Fig. 3 illustrates the learning curves, depicting the relationship between training loss and validation loss in various training scenarios for our proposed models. These curves demonstrate that our models effectively learned the underlying patterns from the datasets during training across different scenarios, with minimal overfitting. From Fig. 3(a) and (b), we can see that the D1 model trains on 80 % of the IQ-OTHNCCD dataset more smoothly than the D2. This might be due to the fact that D2 has more neurons and is prone to overfitting. From Fig. 3(c) and (d), it can be observed that both the D1 and D2 models train on the 10 % of the LC25000 dataset smoothly. However, the D1 learning curve has small spikes from beginning to end, while the D2 learning curve has comparatively big spikes in 2/3 places and is smooth in other places. This might be because there are more neurons in the D2 structure, which requires more data to train on. Again, from 3(e) and 3(f), we can observe that the training of the D1 model on the 80 % of the NCT-CRC-HE-100K dataset as well as the training of the D2 model on the 30 % of the NCT-CRC-HE-100K dataset was very smooth. Nonetheless, the D1 learning cure appears to be more appealing than D2, perhaps because of the low number of training sets for the D2 model. Similarly, Fig. 3(g) and (h) show that training the D2 model on 10 % of the NCT-CRC-HE-100K dataset is comparatively more prone to overfitting than D1. Again, this might be due to the fact that the D2 model has more neurons, requiring more training data.

All of the training in Table 3 was done on the Kaggle platform with free GPU resources, except scenario 1, which was completed on the Google Colab platform with free GPU resources. All the codes were done with Python (with other Python-supported open-source libraries), TensorFlow as the backend, and Keras. We have made all the codes publicly available at the following links.

● Lung Cancer (scenario 1): https://github.com/abdulhasibuddin/Lung-and-Colon-Cancer-Histopathological-Images/blob/main/Lung%20and%20Colon%20Cancer%20Histopathological%20Images/Lung_Colon_Cancer_Hist_10_folds_k1_impl_1_256p_RGB_Dense201_Custom_withImageNet_DataFlow.ipynb
● Lung Cancer (scenarios 5 and 6, visualization for scenario 5): https://www.kaggle.com/code/abdulhasibuddin/lung-cancer-lc25000/notebook
● Lung Cancer (scenarios 2, 3, and 4): https://www.kaggle.com/code/abdulhasibuddin/lung-cancer-iq-oth-nccd/script
● Colon Cancer (scenarios 7, 9, and 10): https://www.kaggle.com/code/abdulhasibuddin/colon-cancer-percent-of-nct-crc-he-100k/notebook
● Colon Cancer (scenarios 8, 11, 12, 13, and 14): https://www.kaggle.com/code/abdulhasibuddin/colon-cancer-nct-crc-he-100k/notebook
● Visualization for IQ-OTHNCCD CT-scan (scenario 2): https://www.kaggle.com/abdulhasibuddin/visualization-lung-cancer-iq-oth-nccd

In general (except scenarios 1, 5, and 6), the training was run with early-stopping set to 5 and the monitor set to validation loss. Therefore, while training, if in five consecutive epochs no improvement in terms of validation loss was encountered, the training would automatically stop. In scenario 1, the number of epochs was set to 5 for all 10-fold training. On the other hand, in scenarios 5 (Fig. 3(c)) and 6 (Fig. 3(d)), the training halted after 122 epochs due to a session timeout on Kaggle.

### 3.4 Statistical metrics

To correctly evaluate the proposed models, we applied the following statistical metrics: Accuracy, Sensitivity/Recall, Specificity, Precision, F1-score, AUC, Jaccard Index, Mathew's Correlation Coefficient, Cohen's Kappa, and Critical Success Index.

**Table:** 

		Predicted Values	
		Positive	Negative
Actual Values	Positive	True Positive (TP)	False Negative (FN)
	Negative	False Positive (FP)	True Negative

Accuracy [40]: It is a measure of the overall correctness of a model's predictions, representing the ratio of correctly predicted instances to the total number of instances.

Accuracy=(TP+TN)/(TP+TN+FP+FN)

It is a general performance indicator but less informative in imbalanced datasets (e.g., rare diseases).

Sensitivity/Recall [40,41]: It indicates how well the model identifies true positives (e.g., correctly diagnosing a disease), which is important in medical settings where missing a positive case can have severe consequences.

Sensitivity=TP/(TP+FN)

It is useful in diagnosing diseases, especially where early detection is critical (e.g., cancer screening).

Specificity [41]: It signifies how well the model avoids false positives (e.g., incorrectly diagnosing a disease), where it is important to minimize unnecessary procedures or treatments.

Specificity=TN/(TN+FP)

It is utilized to ruling out diseases with high costs or risks associated with unnecessary interventions (e.g., biopsies).

Precision [40]: It provides the proportion of predicted positives that are truly positive and is useful when false positives are particularly costly.

Precision=TP/(TP+FP)

It is used in predicting high-risk patients for further investigation (e.g., identifying patients likely to have a specific genetic mutation).

F1-score [40]: It balances precision and recall, providing a single score for overall performance.
F1-score = 2 × Precision × Recall/(Precision + Recall)

It is used when both false positives and negatives are concerning and a compromise between them is desired.

Area Under the Curve (AUC) [40]: It determines the model's ability to discriminate between positive and negative cases. When evaluating overall model performance, especially when comparing different models, AUC is particularly helpful.

Jaccard Index [40]: The Jaccard Index measures the similarity between two sets by dividing the size of their intersection by the size of their union. The Jaccard Index, also known as the Jaccard similarity coefficient or Jaccard similarity index, typically ranges from 0 to 1. A value of 0 indicates no overlap between the sets being compared, meaning they share no common elements, while a value of 1 indicates that the sets being compared are identical, meaning they have complete overlap and share all elements. In a classification task, a Jaccard Index of 0.99 indicates a very high level of similarity between the predicted and true class labels.

JaccardIndex=TP/(TP+FP+FN)

Matthew's Correlation Coefficient (MCC) [42]: MCC takes into account true positives, negatives, and errors, providing a more balanced measure than accuracy, especially in imbalanced datasets.

MCC=(TP×TN−FP×FN)/√((TP+FP)(TP+FN)(TN+FP)(TN+FN))

The significance of MCC increases as the value gets closer to +1 and decreases as the value gets closer to −1. MCC of +1 signifies perfect agreement, when the model perfectly classifies all positive and negative cases and vice versa of −1.

Cohen's Kappa (κ) [43]: The Kappa value implies the inter-rater agreement between the model and a human expert (true label). It assesses how well the model aligns with expert diagnoses.

κ=(Po−Pe)/(1−Pe),

Where.

Po = Accuracy = (TP + TN)/(TP + TN + FP + FN), represents the observed agreement between the raters or classifiers.
Pe = ((TP + FP)(TP + FN) + (TN + FP)(TN + FN))/(TP + TN + FP + FN)^2^, represents the expected agreement under random chance.

A κ value of 1.0 signifies perfect agreement between the model and the human expert when all classifications perfectly match. On the other hand, a κ value < 0.00 signifies poor agreement when the model disagrees with the expert more often than it agrees by chance.

Critical Success Index (CSI) [44]: In medical fields, CSI is used for evaluating models predicting successful patient outcomes or responses to treatment. It measures the accuracy of predictions for events that are rare or infrequent.

CSI=TP/(TP+FP+FN)

A higher CSI (closer to 1) indicates better model performance. This means the model excels at correctly identifying both good and bad cases (e.g., accurately predicting successful treatment outcomes and avoiding false positives for high-risk patients). Contrarily, lower CSI (closer to 0) indicates worse model performance when the model struggles to accurately classify both good and bad cases.

### 3.5 Visualization

We visualized the features from the last convolutional layer of DenseNet201 from scenarios 2 and 5. Class activation maps (CAMs) are a technique to highlight the image regions most influential in the network's decision.

Gradient-weighted Class Activation Mapping (Grad-CAM) [45]: This takes a peek at the final convolutional layer of the neural network. It analyzes the gradients–the rate of change–of the class score (e.g., probability of lung cancer) with respect to the activations in that layer. By averaging these gradients, it creates a heatmap, highlighting image regions that have the strongest influence on the classification. Grad-CAM's simplicity and ease of implementation make it a popular choice.

Grad-CAM++ [46]: This method builds on Grad-CAM, aiming for potentially more precise localization. It incorporates information beyond just the gradients by also considering higher-order derivatives within the network. This can lead to a sharper focus on the most critical image features in the final heatmap visualization.

Score-Weighted Class Activation Mapping (Score-CAM) [47]: This technique ditches the gradients altogether. Instead, it calculates a “score” for each feature map in the final convolutional layer, essentially gauging its importance for the target class (e.g., colon cancer). These scores are then used to create a weighted sum, resulting in a heatmap that emphasizes the most discriminative features for the classification task. Score-CAM can sometimes produce less noisy visualizations compared to Grad-CAM.

Faster Score-CAM [48]: As the name suggests, this method prioritizes efficiency. It uses the core principles of Score-CAM but aims to achieve similar results with less computational power. This can be beneficial for real-time applications or those with limited resources.

CAM Emphasized: In the ‘emphasized’ versions of the CAM visualizations (Grad-CAM emphasized, Grad-CAM++ emphasized, Score-CAM emphasized, and Faster Score-CAM emphasized), we applied a sigmoid function to the heatmap generated by the model. The sigmoid function used in this case is defined as

sigmoid(x,a,b,c)=c/(1+exp(−a*(x−b)))

Where.

x represents the intensity value of a pixel in the CAM heatmap.
a controls the steepness of the sigmoid curve. A higher value of a (set to 50 in this case) leads to a sharper transition between high and low intensity values.
b represents the center point of the sigmoid curve (set to 0.5 here). Values of x closer to b will have a smoother transition through the sigmoid function.
c scales the output of the sigmoid function (set to 1 here). It determines the maximum intensity value achievable after applying the sigmoid.

Applying the sigmoid function with the given parameters to the heatmap values modifies the intensity of the heatmap. Specifically, the sigmoid function scales and compresses the heatmap values between 0 and 1. Higher values in the original heatmap are amplified and pushed towards 1, while lower values are compressed towards 0.

This process effectively enhances the contrast and emphasizes the regions in the heatmap where the model is more confident about the presence of the target class. As a result, areas with higher activation scores in the original heatmap are highlighted more prominently in the CAM visualization, making it easier to interpret and visualize the areas of interest for the given class.

While all these visualization techniques offer valuable insights, their strengths can be situation-specific. For a balance of simplicity and interpretability, Grad-CAM is a good starting point. If potentially more precise localization is needed, Grad-CAM++ might be worth exploring. Score-CAM can be a good option when looking for potentially less noisy visualizations. Faster Score-CAM is useful when computational efficiency is a top priority.

## 4 Results and discussions

The data presented in Table 5 illustrates the evaluation of the D1 model's performance using a 10-fold cross-validation method with five epochs. Each fold represents a different data split for training and testing.

**Table 5:** Summary of 10-fold cross-validation for applying the proposed D1 model to the LC25000 dataset.

Fold	Accuracy	Sensitivity/Recall	Specificity	Precision	F1-Score	AUC
1	1.0	1.0	1.0	1.0	1.0	1.0
2	0.9996	0.9996	0.9999	0.9996	0.9996	1.0
3	0.9992	0.9992	0.9998	0.9992	0.9992	0.9997
4	1.0	1.0	1.0	1.0	1.0	1.0
5	0.9992	0.9992	0.9998	0.9992	0.9992	1.0
6	1.0	1.0	1.0	1.0	1.0	1.0
7	1.0	1.0	1.0	1.0	1.0	1.0
8	1.0	1.0	1.0	1.0	1.0	1.0
9	0.9984	0.9984	0.9996	0.9984	0.9984	1.0
10	0.9996	0.9996	0.9999	0.9996	0.9996	1.0
Avg	0.9996	0.9996	0.9999	0.9996	0.9996	0.99997 (∼1.0)

Based on the information provided in Table 6, the performance comparison of different methods for cancer classification is as follows.

● Using the Enhanced Grasshopper Optimization Algorithm (EGOA), Pradhan and Sahu [7] achieved an accuracy of 98.50 % for lung cancer classification.
● Ram et al. [8] employed a method called GS-PCANet and attained an accuracy of 90.80 % for lung cancer classification, with an AUC (a measure of accuracy) of 0.95.
● Reis and Turk [9] utilized DenseNet169 for colon cancer classification and achieved an accuracy of 95.0 %.
● Sethy et al. [12] combined the AlexNet architecture, wavelet transformations, and support vector machines to achieve an accuracy of 99.3 % and an impressive AUC of 0.99 for lung cancer classification on the LC25000 dataset.
● Hadiyoso et al. [13] employed a CNN with the VGG16 architecture and CLAHE to achieve an accuracy of 98.96 % for lung cancer classification.
● Rajput and Subasi [11] used ResNet50 and achieved a remarkable accuracy of 99.8 % for colon cancer classification, although they did not report AUC metrics.
● Iqbal et al. [15] introduced ColonNet for colon cancer classification, achieving an accuracy of 96.31 % along with an AUC of 0.95 using histopathology images as the dataset.

**Table 6:** Performance comparison of the proposed D1 method against state-of-the-art works.

Author [reference]	Year	Dataset	Cancer class	Method	Performance	
					Accuracy (%)	AUC
Pradhan and Sahu [7]	2023	LC25000	Lung cancer	Enhanced grasshopper optimization algorithm (EGOA)	98.50	–
Ram et al. [8]	2023	Histopathology images	Lung cancer	GS-PCANet	90.80	0.95
Reis and Turk [9]	2023	MNIST	Colon cancer	DenseNet169	95.00	–
Rajput and Subasi [11]	2023	Histopathology images	Colon cancer	ResNet50	99.80	–
Sethy et al. [12]	2023	LC25000	Lung cancer	AlexNet, wavelet, and support vector machines	99.30	0.99
Hadiyoso et al. [13]	2023	LC25000	Lung and colon cancer	CNN with Contrast Limited Adaptive Histogram Equalization (CLAHE) and VGG16 architecture	98.96	–
Iqbal et al. [15]	2023	LC25000	Lung and colon cancer	ColonNet	96.31	0.95
Stephen and Sain [17]	2023	LC25000	Lung and colon cancer	Gaussian processes are used in a Bayesian convolutional neural architecture search technique	93.91	–
Kumar et al. [18]	2022	LC25000	Lung and colon cancer	DenseNet-121	98.60	–
Hage Chehade et al. [19]	2022	LC25000	Lung and colon cancer	XGBoost, SVM, RF, LDA, MLP and LightGBM	99.00	–
Mehmood et al. [21]	2022	LC25000	Lung and colon cancer	AlexNet	98.40	–
Talukder et al. [24]	2022	LC25000	Colon and lung cancer	Model for extracting hybrid ensemble features	99.30	–
Adu et al. [27]	2021	LC25000	Colon and lung cancer	Network of dual horizontal squash capsules	99.23	–
Karim et al. [29]	2021	LC25000	Lung and colon cancer	Double CLAHE, Deep Learning (DL) Algorithms and Artificial Intelligent	98.15	–
Proposed method (D1)	2023	LC25000	Lung and colon cancer	Proposed Dense network	99.96	0.99997

In summary, the proposed D1 method demonstrated exceptional performance in both colon and lung cancer classifications using the proposed dense network, achieving an accuracy of 99.96 % and a perfect AUC of 1.00.

The proposed D1 dense network outperforms other techniques in terms of precision for both lung and colon cancer categorizations, as evidenced by the provided results. The fact that it achieves a flawless AUC further highlights its superior ability to differentiate between positive and negative cases.

The presented results indicate that the proposed model has achieved outstanding performance across multiple evaluation metrics. The implications and potential reasons for these results are as follows.

● First, the accuracy of 0.9996 suggests that the model's predictions are correct in the majority of cases, indicating successful learning of data patterns and accurate predictions.
● The sensitivity and recall values of 0.9996 demonstrate the model's effectiveness in identifying positive instances with a low rate of false negatives, which is crucial in scenarios such as disease detection and fraud prevention.
● The high specificity values of 0.9999 indicate a low number of false positives, which is beneficial, especially in situations where the cost of false positives is significant, such as cancer detection.
● The model's high precision value of 0.9996 suggests that it is highly accurate when predicting positive instances, particularly important in scenarios with costly false positives, like medical diagnoses.

The F1-score provides a balanced assessment of the model's performance by combining precision and recall. With an average value of 0.9996, the model successfully strikes a solid balance between precision and recall, guaranteeing excellent accuracy and a low rate of false positives and false negatives.

Furthermore, the remarkable AUC value of 0.99997 (approximately 1.0) underscores the model's exceptional proficiency in discriminating between positive and negative instances across a spectrum of probability thresholds. This outstandingly high AUC signifies that the model excels at prioritizing positive instances by consistently assigning them significantly higher probabilities than negative instances, reaffirming its robust predictive capabilities. A concise overview of how the suggested models performed across various scenarios, along with comparisons to benchmarks and relevant interpretive remarks, is provided in Table 7. On the other hand, precision, recall, F1-score per class, and their associated supports when evaluating the suggested models across a range of test sets in varying scenarios are given in Table 8, while Table 9 provides at a glance a summary of the Jaccard Index, Matthew's Correlation Coefficient, Cohen's Kappa, and Critical Success Index values.

**Table 7:** A brief summary of the proposed models’ performances in all of the applied scenarios, corresponding benchmark comparisons, and respective interpretive comments.

Scenario	Feature Extraction (ImageNet Pre-trained)	Epoch	Test Accuracy (%)	Benchmark Accuracy (%)	Comments
1	DenseNet201	05	99.96	98.50 (2023) [7], 99.30 (2023) [12], 98.96 (2023) [13]	D1 performs better than the benchmarks, proving its superiority.
2		55	92.24	93.21 (2023) [49], 86.42 (2022) [50], 88.00 (2021) [51]	Both D1 and D2 significantly outperform Chen et al. [51] and Bangare et al. [50], while the ensemble performs parallelly to Mohamed et al. [49]. This proves the multi-modality and robustness of D1 and D2.
3		53	91.74		
4		n/a	93.00		
5		122	99.19	98.50 (2023) [7], 99.30 (2023) [12], 98.96 (2023) [13]	With only 10 % images of the dataset, both the D1 and D2 models either outperform or perform parallelly to the benchmarks. This proves the efficiency of D1 and D2.
6		122	99.30		
7		20	99.80	94.80 (2023) [52], 96.26 (2023) [53], 96.16 (2021) [54], 96.00 (2020) [55]	From 80 % to as little as 10 %, training data did not affect the performances of D1 and D2, compared to the benchmarks. This proves the efficiency and robustness of D1 and D2.
8		50	99.53		
9		53	95.00		
10		110	96.00		
11	ResNet50	09	82.98	59.89 (InceptionV3), 61.11 (Xception), 64.33 (DenseNet201)	Even with an imbalanced training dataset containing a very low number of images, both D1 and D2 achieved more than 80 % accuracy on the large 100K test set, whereas all the benchmarks were significantly lower. This proves the resilience and robustness as well as the efficiency of D1 and D2.
12		17	82.37		
13		11	82.89		
14		13	83.26

**Table 8:** Class-wise precision, recall, f1-score, and corresponding supports for testing the proposed models on diverse test sets in different scenarios. The best scores are provided in bold.

Test Set	Class	Model 1 (D1)					Model 2 (D2)				
		Scenario	Precision	Recall	F1-score	Support	Scenario	Precision	Recall	F1-score	Support
LC25000	Lung_N	5	**1.00**	**1.00**	**1.00**	4500	6	**1.00**	**1.00**	**1.00**	4500
	Lung_ACA	5	**1.00**	**1.00**	**1.00**	4500	6	**1.00**	**1.00**	**1.00**	4500
	Lung_SCC	5	**1.00**	**1.00**	**1.00**	4500	6	**1.00**	**1.00**	**1.00**	4500
	Colon_N	5	0.98	**0.98**	**0.98**	4500	6	**0.99**	**0.98**	**0.98**	4500
	Colon_ACA	5	**0.98**	0.98	**0.98**	4500	6	**0.98**	**0.99**	**0.98**	4500
NCT-CRC-HE-100K	ADI	7	**1.00**	**1.00**	**1.00**	2081	8	**1.00**	**1.00**	**1.00**	7284
	BACK	7	**1.00**	**1.00**	**1.00**	2113	8	**1.00**	**1.00**	**1.00**	7396
	DEB	7	**1.00**	**1.00**	**1.00**	2302	8	**1.00**	0.99	0.99	8058
	LYM	7	**1.00**	**1.00**	**1.00**	2311	8	**1.00**	**1.00**	**1.00**	8089
	MUC	7	**1.00**	**0.99**	**1.00**	1779	8	**0.99**	**0.99**	**0.99**	6227
	MUS	7	**1.00**	**1.00**	**1.00**	2707	8	**1.00**	0.99	**1.00**	9475
	NORM	7	**1.00**	**1.00**	**1.00**	1752	8	**1.00**	0.99	0.99	6134
	STR	7	**1.00**	**1.00**	**1.00**	2089	8	0.98	0.99	0.99	7312
	TUM	7	**1.00**	**1.00**	**1.00**	2863	8	0.99	0.99	0.99	10021
CRC-VAL-HE-7K	ADI	9	**1.00**	0.97	**0.99**	1338	10	**1.00**	**0.98**	**0.99**	1338
	BACK	9	**1.00**	**1.00**	**1.00**	847	10	**1.00**	**1.00**	**1.00**	847
	DEB	9	**0.91**	**0.97**	**0.94**	339	10	**0.91**	0.96	0.93	339
	LYM	9	**0.99**	0.97	0.98	634	10	**0.99**	**0.99**	**0.99**	634
	MUC	9	0.93	**1.00**	0.96	1035	10	**0.96**	0.98	**0.97**	1035
	MUS	9	0.81	0.88	0.84	592	10	**0.82**	**0.89**	**0.85**	592
	NORM	9	0.92	**0.99**	**0.96**	741	10	**0.95**	0.97	**0.96**	741
	STR	9	**0.92**	0.67	**0.78**	421	10	0.86	**0.72**	**0.78**	421
	TUM	9	**0.99**	0.95	**0.97**	1233	10	0.98	**0.97**	**0.97**	1233
IQ-OTHNCCD	BN	2	**0.85**	**0.46**	**0.59**	24	3	0.82	0.38	0.51	24
	MG	2	**1.00**	0.98	0.99	112	3	**1.00**	**0.99**	**1.00**	112
	N	2	**0.84**	**0.98**	**0.91**	83	3	**0.84**	**0.98**	0.90	83

**Table 9:** Summary of all the Jaccard Index (J), Matthew's Correlation Coefficient (MCC), Cohen's Kappa (Kp), and Critical Success Index (CSI) values for this study.

Dataset	Jaccard Index (J)	Matthew's Correlation Coefficient (MCC)	Cohen's Kappa (κ)	Critical Success Index (CSI)
LC25000 (k1)	1	1	1	1
LC25000 (k2)	0.9996	0.9996	0.9836	0.9992
LC25000 (k3)	0.9984	0.9976	0.9675	0.9984
LC25000 (k4)	1	1	1	1
LC25000 (k5)	0.9984	0.9976	0.9675	0.9984
LC25000 (k6)	1	1	1	1
LC25000 (k7)	1	1	1	1
LC25000 (k8)	1	1	1	1
LC25000 (k9)	0.9968	0.9952	0.9694	0.9968
LC25000 (k10)	0.9996	0.9996	0.9836	0.9992

**Scenario**	**J**	**MCC**	**κ**	**CSI**
1	0.9993	0.9987	0.9853	0.9990
2	0.7439	0.8689	0.8639	0.7020
3	0.7184	0.8614	0.8551	0.6650
4	0.7556	0.8839	0.8796	0.7140
5	0.9840	0.9898	0.9898	0.9837
6	0.9863	0.9913	0.9913	0.9861
7	0.8371	0.9073	0.9057	0.8213
8	0.9906	0.9946	0.9946	0.9906
9	0.8845	0.9455	0.9452	0.8745
10	0.8926	0.9504	0.9503	0.8798
11	0.7227	0.8095	0.8081	0.6671
12	0.7199	0.8027	0.8013	0.6563
13	0.7197	0.8081	0.8070	0.6616
14	0.7265	0.8129	0.8113	0.6765

Now, from Table 6, we can also observe that several other studies have achieved almost similar results, which are above 99 % accuracy. However, the proposed models in this paper have some benefits over the existing ones. While the works mentioned in Table 6 are only applicable to histopathological images, those studies are not tested on multiple types of images, require significant pre-processing, and use a large amount of data while training. In contrast, our proposed models can perform in parallel on multiple types of images with imbalanced classes and require a fraction of the original datasets (Table 7). In summary, this study has made the following contributions.

1. Development of two different DNN architectures (D1 and D2), utilizing transfer learning through ImageNet pre-trained DenseNet201 for feature extraction, with the exception of using ImageNet pre-trained ResNet50 for feature extraction in models trained on the CRC-VAL-HE-7K dataset.
2. Application of the D1 and D2 models to four different datasets: NCT-CRC-HE-100K (colon cancer), CRC-VAL-HE-7K (colon cancer), LC25000 (lung and colon cancer), and IQ-OTHNCCD (lung cancer). These datasets were selected to demonstrate the multi-modality, resiliency, and efficiency of our proposed models in classifying colon and lung cancers from images. In general, the models were trained until they reached a point of no further improvement in validation loss for five consecutive epochs.
3. Three of the datasets (NCT-CRC-HE-100K, CRC-VAL-HE-7K, and LC25000) contain histopathological images, while the IQ-OTHNCCD dataset contains CT scan images, highlighting the multi-modality of the developed models.
4. Acknowledgement of the imbalanced nature of some datasets, with particular emphasis on the high imbalance in the CRC-VAL-HE-7K and IQ-OTHNCCD datasets, showcasing the resilience and robustness of our proposed models.

To provide an overall performance summary of our proposed models.

● The D1 model was trained on 80 % of the NCT-CRC-HE-100K dataset (80,000 images) and achieved 99.80 % accuracy.
● 10-fold cross-validation on the LC25000 dataset with the D1 model resulted in an average validation accuracy of 99.96 %, surpassing recent performances reported by Pradhan and Sahu [7], Sethy et al. [12], Hadiyoso et al. [13], and others.
● Both the D1 and D2 models were trained on 80 % of the IQ-OTHNCCD dataset (878 images) and achieved accuracies of 92.24 % and 91.78 %, respectively, with an ensemble performance of 93 %. These results surpassed recent performances reported by Chen et al. [51] and Bangare et al. [50] and were on par with performances reported by Mohamed et al. [49] and Al-Yasriy et al. [56].

To assess the efficiency of the developed architectures, we considered the following scenarios.

● The D1 and D2 models were trained on 10 % of the randomly chosen (2,500) images from the LC25000 dataset for 122 epochs each, achieving accuracies of 99.19 % and 99.30 %, respectively. These performances outperformed those reported by Pradhan and Sahu [7], Hadiyoso et al. [13], Kumar et al. [18], and others.
● The updated version of the D1 model (D2 model) was trained on 30 % of the randomly chosen (30,000) images from the NCT-CRC-HE-100K dataset for 50 epochs, achieving 99.53 % accuracy.
● Both the D1 and D2 models were trained on 10 % of the randomly chosen (10,000) images from the NCT-CRC-HE-100K dataset for 53 and 110 epochs, respectively, and tested on the CRC-VAL-HE-7K dataset. The accuracy achieved was 95 % and 96 %, respectively, surpassing the results reported by Sun et al. [52] and Kather et al. [57], while the D2 model performed comparably to the results reported by Kumar et al. [53], Liang et al. [55], and Ghosh et al. [54].
● The D2 model trained on the CRC-VAL-HE-7K dataset (7180 images) for 9 epochs and tested on the NCT-CRC-HE-100K dataset (100,000 images) significantly outperformed all benchmarks, achieving an accuracy of 82.98 %.
● The D2 model trained on the balanced-downsampled version of the CRC-VAL-HE-7K (CRC-7K-Down) dataset (3051 images) for 17 epochs and tested on the NCT-CRC-HE-100K dataset achieved notably higher performance (82.37 % accuracy) compared to the benchmark performance of training the Xception model on the same dataset.

It was observed that in the NCT-CRC-HE-100K and CRC-VAL-HE-7K datasets, images from the smooth muscle (MUS) and cancer-associated stroma (STR) classes posed the greatest challenge for correct identification. To address this, in the CRC-7K-Down dataset, the number of images corresponding to these two classes was tripled using the Sampling With Repetition (SWR) approach (CRC-7K-Down-MUS.STRx3 dataset). Both the D1 and D2 models were trained on this dataset for 11 and 13 epochs, respectively, and tested on the NCT-CRC-HE-100K dataset. Additionally, the Xception model was trained to create a benchmark for the dataset. Both of our proposed models significantly outperformed the benchmark performance, with accuracies of 82.89 % and 83.26 %, respectively.

**Fig. 4:**
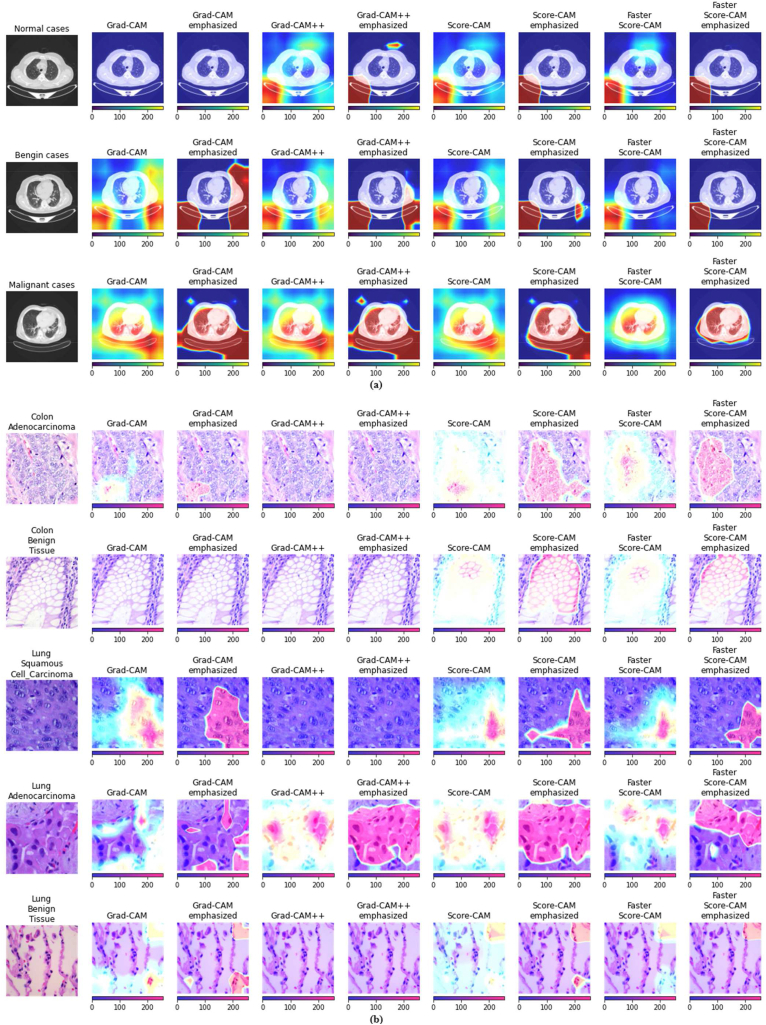
Feature visualization for the last layer (after dropping the top layer) of DenseNet201 from (a) the IQ-OTHNCCD (Iraq-Oncology Teaching Hospital/National Center for Cancer Diseases) and (b) the LC25000 (Lung and Colon Cancer Histopathological Image) datasets for scenarios 2 and 5, respectively. Here, the first column provides original samples from each class of the two datasets. The subsequent columns present the Grad-CAM, Grad-CAM++, Score-CAM, and Faster Score-CAM, with Grad-CAM emphasized, Grad-CAM++ emphasized, Score-CAM emphasized, and Faster Score-CAM emphasized, respectively.

● To better understand the nature of the extracted features from ImageNet-pretrained CNN models, we visualized several layers from the ImageNet-pretrained DenseNet201 architecture (Fig. 4). The model comprises several layers with a high number of units or neurons, including dense layers with 1024 and 2048 units. This deep and wide design allows the model to learn hierarchical representations and understand complex interactions, enhancing its ability to handle intricate data.

The reasons behind our proposed models performing up to par in all scenarios can be described as follows.

● Transfer Learning: The utilization of pre-trained models like DenseNet201 and ResNet50 enables the extraction of high-level features from images. These architectures have learned representations of general visual features from a large and diverse dataset (ImageNet). This often leads to better performance on downstream tasks compared to training from scratch.
● Adaptability: The subsequent dense layers (Dense 1, Dense 2, and Dense 3) allow for fine-tuning the learned features to better fit the specifics of the target dataset. Adjusting the number of neurons and layers gives flexibility to capture dataset-specific patterns and nuances.
● Regularization: The inclusion of L2 regularization and dropout in the dense layers helps prevent overfitting. Regularization techniques like L2 regularization impose penalties on large weights, encouraging the model to generalize better. Dropout randomly deactivates a fraction of neurons during training, reducing co-dependencies among neurons and improving generalization.
● Activation Function: ELU (Exponential Linear Unit) as the activation function in the dense layers helps alleviate the vanishing gradient problem, allowing for better learning and convergence.
● Model Complexity: The variations in the number of neurons and layers between D1 and D2 might capture different complexities in the data. This flexibility could allow the models to adapt to different levels of intricacy in the dataset, potentially capturing more nuanced patterns.
● Softmax Output: The use of a softmax activation function in the output layer for multi-class classification ensures that the model generates class probabilities, aiding in more confident and accurate predictions.

Overall, the combination of transfer learning, fine-tuning, regularization, and adaptable model complexities seems to contribute to the superior performance of models D1 and D2 compared to benchmark models. Nonetheless, while the results highlight their potential for significant advancements in medical diagnostics and early cancer detection, further discussion on the practical clinical use of these models, including integration into existing workflows, validation in clinical trials, regulatory approval, scalability, and potential challenges in deployment, would enhance understanding of their real-world impact and implementation in healthcare settings.

We display the visualizations corresponding to this study in Fig. 4. In Fig. 4(a), we can see that in ‘normal’ cases, barely any regions within the body part of the CT-scan images got any attention. On the other hand, in ‘benign’ cases, some parts of the body region received attention, indicating minor infections. In contrast, for the ‘malignant’ cases, the entire body region of the internal organs also got attention, indicating serious infections. We acknowledge that the CT scans were acquired from different machine setups. However, it's crucial to note that images from all machine setups were represented across all three classes (malignant, normal, and benign), and they were distributed between the training and test sets. As a result, any potential bias stemming from machine-setup differences is mitigated, ensuring that it's unlikely to significantly impact the final results.

Again, from the visualizations of the histopathological lung cancer images in Fig. 4(b), we observe that the benign tissue images received the least attention. On the other hand, in the adenocarcinoma image, the infected glandular parts and in the squamous cell carcinoma image, the infected areas of the squamous flat cell got attention. In this particular case, we can see that the adenocarcinoma visualization indicates more infection compared to the squamous one.

Moreover, from the visualizations of the histopathological colon cancer images in Fig. 4(b), we can perceive that for the ‘benign’ case, the visualizations indicate minor infection, while for the ‘adenocarcinoma’ case, the visualizations indicate large infection regions.

## 5 Conclusion and future work

This study introduces two innovative deep neural network architectures, D1 and D2, which leverage transfer learning from pre-trained models to classify various types of cancer across multiple datasets, encompassing histopathological and CT scan images. These models exhibit remarkable resilience and efficiency, consistently achieving high accuracies in diverse scenarios and datasets. Particularly noteworthy is their ability to surpass existing benchmarks, excelling in addressing challenges posed by imbalanced datasets and difficult-to-classify categories such as smooth muscle and cancer-associated stroma. Employing careful training strategies, including ensemble learning and data augmentation techniques such as sampling with repetition, these models consistently demonstrate superior performance, underscoring their potential for robust cancer classification across a wide range of imaging datasets.

While existing studies achieve high accuracy on specific types of cancer images, our proposed models offer several advantages: they work on multiple image types and imbalanced datasets with minimal preprocessing, requiring significantly less data for training and achieving similar performance.

Nonetheless, there are several limitations to this work, which can be described as follows.

● In terms of multi-modality, we have trained the models on multiple image types (histopathological and CT-scan) of lung cancer as well as multiple cancer types (lung and colon). The models could also be trained on multiple types of images of colon cancer. Also, other cancer types could be tested using the proposed models.
● In terms of robustness, this study addresses this issue from only a balanced-imbalanced perspective. Other perspectives (noise, image size, and resolution) should also be addressed.
● In terms of efficiency, while the models are capable of generalizing on the cancer images even with a fraction of the datasets, efficiency from the perspectives of memory and GPU resources as well as training time should also be determined.

In the future, our focus can shift towards addressing the limitations of this work. Firstly, to enhance the model's versatility, future studies could train the models on various image types of colon cancer and potentially even incorporate additional data modalities like patient information for a more comprehensive analysis. Additionally, testing the models on a wider range of cancer types would assess their generalizability.

Secondly, to ensure the model's robustness in real-world scenarios, future research should evaluate its performance under diverse conditions. This includes introducing various types of noise into the data, testing images with varying sizes and resolutions, and investigating its susceptibility to adversarial attacks. Implementing appropriate techniques can improve the model's ability to handle such challenges.

Finally, to optimize efficiency, future studies should analyze both memory and GPU resource usage during training and inference. Exploring techniques like model compression and pruning can potentially reduce resource requirements. Additionally, investigating training time optimization and utilizing pre-trained models hold promise for improving efficiency without compromising accuracy.

By addressing these limitations through further research and development, we can strive towards a more robust, efficient, and widely applicable approach for cancer detection and diagnosis using deep learning models.

## Ethical approval

The data sources utilized in our analysis were obtained from a publicly accessible repository. It is important to note that our research did not involve any experiments involving human or animal participants. Approval from the ethics committee is unnecessary.

## Data availability statement

No data was used for the research described in the article.

## CRediT authorship contribution statement

**A. Hasib Uddin:** Writing – review & editing, Validation, Project administration, Methodology, Formal analysis, Conceptualization. **Yen-Lin Chen:** Writing – review & editing, Writing – original draft, Funding acquisition. **Miss Rokeya Akter:** Visualization, Resources. **Chin Soon Ku:** Writing – review & editing, Validation, Funding acquisition. **Jing Yang:** Investigation, Data curation. **Lip Yee Por:** Writing – review & editing, Validation, Project administration, Conceptualization.

## Declaration of competing interest

The authors declare that they have no known competing financial interests or personal relationships that could have appeared to influence the work reported in this paper.
